# The long arm of repression: determinants of psychotherapy use among East Germans and its relevance for today’s institutional trust—psychotherapeutic implications of political repression in the former German Democratic Republic

**DOI:** 10.3389/fpubh.2025.1601917

**Published:** 2025-07-22

**Authors:** Marie-Theresa Kaufmann, Hannah D. Nussmann, Ayline Heller, Christoph Kasinger, Elmar Brähler, Adrian Gallistl, Bernhard Strauß

**Affiliations:** ^1^Institut für Psychosoziale Medizin, Psychotherapie und Psychoonkologie, Universitätsklinikum Jena, Jena, Germany; ^2^Fachbereich für Angewandte Sozialwissenschaften, Fachhochschule Dortmund, Dortmund, Germany; ^3^Survey Design and Methodology, GESIS – Leibniz-Institut für Sozialwissenschaften eV, Mannheim, Germany; ^4^Klinik und Poliklinik für Psychosomatische Medizin und Psychotherapie, Johannes Gutenberg-Universität Mainz, Mainz, Germany; ^5^Abteilung für Medizinische Psychologie und Medizinische Soziologie, Universität Leipzig, Leipzig, Germany

**Keywords:** psychotherapy, repression, system change, GDR, institutional trust

## Abstract

**Introduction:**

Maintaining trust in social institutions is a critical challenge for Western democracies. We examine the role of psychotherapy on institutional trust in the former German Democratic Republic (GDR; now: New Federal States of Germany) which used open and covert methods to keep opposition members under control.

**Methods:**

The study with *n* = 1,805 individuals who were born and socialized in the former GDR (i.e., born before 1980) was conducted in 2022. Logistic regression models to predict a person’s probability of psychotherapy use after the system change from the GDR to the New Federal States of Germany were built using a basic model derived from the literature with predictor variables such as gender and education. This model was extended by experiences of repression. In a second analysis, linear regression models to predict institutional trust were analyzed following a similar strategy with the addition of psychotherapy experience as a predictor.

**Results:**

Reporting repression in the GDR (44% of the total sample) was related to a higher probability of psychotherapy use. In the group who reported personally experienced repression (15% of the total sample), psychotherapy appeared to be relevant for higher levels of institutional trust.

**Discussion:**

Psychotherapy might have the potential to help regaining institutional trust after a system change. Psychotherapists should consider that patients who experienced (post-)socialism were commonly affected by repression and might show less institutional trust including the healthcare system. Furthermore, this study revealed an estimation of the occurrence of repression in a representative sample in the former GDR.

## 1 Introduction

People who were socialized in the former German Democratic Republic (GDR; 1949–1989; now: New Federal States of Germany/East Germany), now living in the reunified country, face the reality of having experienced two different political systems. Socialization in the GDR with a dictatory regime and immediately following the dictatorship of the National Socialism seems to be connected with a set of specific stressors. This may also affect different views as for instance the layman concepts of psychiatric disorders (1). It is likely that these experiences and views lead to differences in the need for and the use of psychotherapy. In this study, we investigate the relationship between GDR socialization and psychotherapy use as well as current institutional trust.

Previous international studies identified differences in psychotherapy use between different groups of people. People with white skin color, in multi-ethnic cultures, women, people with higher education, and people between the ages of 21 and 50 have the highest levels of psychotherapy experience. Those who are divorced or separated, have never been married, and have white skin are most likely to be willing to go to therapy after getting to know the therapist in a first meeting (2). Men and people with lower education mention more negative attitudes towards psychotherapy (3, 4). The education factor seems to have a different effect when comparing women and men in Germany: Women with higher educational qualifications report less feelings of shame when seeking psychotherapy. Contrary, men with higher educational levels report more feelings of shame when seeking psychotherapy (5). It is estimated that almost 16% of the adult German population have already received psychotherapeutic or psychiatric treatment (6). Overall, more people with mental illness seem to seek help in recent years in Germany (7, 8). East Germans prefer psychopharmacological treatment to psychotherapeutic treatment for a mental illness (6). Ignorance, fear of stigmatization, severe mental illness, living in East Germany, low personal psychological strain and low socioeconomic level are known as impeding factors seeking psychotherapeutic help (7, 9–13). The choice of psychotherapeutic options in Germany is determined by the place of residence and patient characteristics: YAVIS (‘young, attractive, verbal, intelligent, successful’), are overrepresented and HOUND (‘humble, old, unattractive (unsuccessful), nonverbal (not social), dumb’) patients are underrepresented (13). Taken together, it is likely that certain experiences of GDR-Socialization might be related to psychotherapy usage as living in East Germany is an already identified factor in psychotherapy use.

The Ministry for State Security of the GDR (`Ministerium für Staatssicherheit’; commonly abbreviated as `Stasi’) itself was interested in finding mentally healthy employees, as a research paper on days of incapacity for work from the 1970s shows at the ‘Law School’ (‘Juristische Hochschule’; JHS)—the central educational and research institution of the ‘Stasi’ which was unknown to most citizens (14)—in the ‘Stasi Records Archive’ [‘Stasi-Unterlagen-Archiv’, BStU; (15) - a governmental institution - shows and was critical here]. Ideally, psychotherapeutic treatment should have been provided by its own staff, but this was lacking (15). There is little data on the use of psychotherapy during the GDR, which indicates low case numbers: A psychotherapy association of the GDR carried out a representative survey in a GDR city in 1981: Of the 3,000 participants, 465 people had an indication for psychotherapy and only 46 (<10% of participants with an indication) received such treatment (16). To what extent psychotherapy was an option for mental stress in the GDR is questionable.

In the GDR, the state used open and covert methods to keep opposition members under control: From its very beginning, the Soviet Government Agency (‘komitet gossudarstwennoi besopasnosti’; KGB) and the ‘Stasi’ used hard repression, such as imprisonment with potentially traumatic experiences such as torture, as well as soft and non-physical forms of repression such as observation. After signing the Declaration of Helsinki in 1975, the secret police had to use more subtle strategies of repression such as (school) career slumps. Using several forms of soft repression to weaken and defeat enemies of the state is subsumed under the term disintegration [‘Zersetzung’, (17)], using a systematic network of observation, including inofficial members (IM) of the ‘Stasi’, who controlled all social areas with an observation density of at least one IM per 165 in Berlin (East) up to 84 in Cottbus inhabitants in the year 1988 (18, 19). Borbe (20) reported that estimates of political prisoners and victims of the SED regime in the GDR range from a minimum of 170,322 (including 322 killed for political reasons) to a maximum of 5.828 million, which includes those who voluntarily left the GDR. In a non-representative sample, almost 40% of GDR citizens who were imprisoned for political reasons, as well as their partners and children reported use of psychological treatment. Family members also often experienced additional forms of repression—especially during the period of imprisonment (21).

The various consequences of injustice on a psychological level are undisputed: Experiences of hard repression as imprisonment often lead to multiple psychiatric symptoms, such as anxiety or PTSD (22). Posttraumatic stress disorder symptoms were shown to be weaker when individuals experienced greater social support (23, 24). Less support was associated with more silence about the traumatic content among persons who were affected by repression (25). Individuals who experienced soft repression not leading to criminal prosecution were also affected with prevalence rates of 60–70% for mental illness, mainly affective, anxiety and somatoform disorders (26, 27). Transgenerational effects were evident in the study of Klinitzke et al. (28), in which children of imprisoned parents showed significantly higher psychological stress—with no difference in the time of birth (before/after the parents’ imprisonment). There are currently, few data from representative population surveys on the number of people affected by SED injustice, who often have a higher level of psychological stress and therefore a greater need for psychotherapy. One example is the Thuringian Social Study 2022 (29), which is only carried out regionally and not in all regions of East Germany: In the year 2022 almost 13% indicated having personally experienced injustice.

In the GDR, the ‘Stasi’ and its (inofficial) members seemed to try gaining trust (30–32). It is known that observation density, a soft form of repression, still is connected to today’s interpersonal trust, institutional trust levels and economic outcomes (33). International research shows that institutional trust influences interpersonal trust (34). Furthermore, institutional trust can be separated into political trust as in the government, trust in impartial institutions as the police or the healthcare system and trust in control institutions (e.g., the media). Interestingly, the healthcare system usually shows highest scores of trust (35). Baroudi et al. (36) found that women have less trust in the healthcare system and older people have higher levels of trust. People with more community involvement and those with lower incomes report higher levels of trust, while those with less education, of foreign origin, or with economic stress report lower levels. Institutional trust currently seems to be similar (and high in comparison to previous decades) among East and West Germans (37). Following the first decade after the reunification, institutional trust was low in both West and East Germany. This was mainly due to a decrease in institutional trust in West Germany and a slight increase in East Germany (38). After a slight increase in both regions of Germany, East German values approached to West German trust values in institutions (39). Previous models showed significant associations of institutional trust values in Germany with sociodemographic factors such as gender, high school diploma, age (37) and income (40), but also political aspects like voting behavior, the reunification process itself or civic engagement (37, 40). Feeling as a winner or loser of the reunification has an impact with perceived losers showing smaller trust values (37). A high trust level in institutions seems to be important for psychological wellbeing (41), social cohesion (42) and it can have a buffer function between adversities and wellbeing (43, 44). With regard to social processes, institutional trust also seems to be important for the economic management of a country (33) and is necessary for governments to manage crises at a national level (45). Among AIDS patients, higher levels of trust in physicians and institutions have been shown to be associated with more favorable disease outcomes (46). This finding may have implications for psychotherapeutic treatment, too. Consequently, institutional trust is important both on a personal and global societal level.

### 1.1 Research questions

Trying to connect questions related to the use of psychotherapy among East Germans and the construct of institutional trust, this study explores the following questions: First, who uses psychotherapy in the New Federal States (former GDR)? We argue that experiences of repression are related to the use of psychotherapy. To this end, we investigated data from people living in East Germany, who were socialized in the GDR. Next to the above-mentioned well-known factors that are associated with the use of psychotherapy (female gender, higher education, not living together with a partner and receiving higher income) we added psychological burden (anxiety, depressive and somatoform symptoms) which should be associated with higher psychotherapy use. Furthermore, we added repression. This allowed us to initially assess the social function of psychotherapy in a society that was affected by repression.

Second, we evaluate the role of psychotherapy in regaining trust in the New Federal States. To this end, we exploratorily investigated the following question: Is there a connection between psychotherapy experience and institutional trust in East Germany? Based on the literature, a basic model was tested with sociodemographic factors (female gender, higher education, being married and living together, income) and political factors (civic engagement, voting behavior, experience of the reunification). In addition, the binary variable of psychotherapy use (yes/no) was added. The relationships between the variables will be tested taking the relevance of repression on psychotherapy use into account.

## 2 Methods

### 2.1 Study design, setting and participants

We carried out a cross-sectional general-population study in the New Federal States of Germany between May and September 2022—performed by the independent pollster research institute USUMA (‘Unabhängige Serviceeinrichtung für Umfragen, Methoden und Analysen’) located in Berlin. The entire survey time took almost 70 min with a main focus on biographies in East Germany, encompassing e. g. the reunification process or unemployment. USUMA is a member of the ADM F2F (face-to-face, oral-written) working group. In accordance with the ADM standard, the procedure can be assessed as a representative random sample. Based on accessible data such as the municipality structure of Germany, an area sample was first created, which was then stratified into sampling points. A target household was picked using the random route method and a target person in the household was selected following the Kish-Selection-Grid method [ADM, accessed on 25.04.2024; (47)]. People aged 16 and over were eligible to participate. The response rate was 45%. Individuals who were not born in East Germany and two persons who filled out the wrong part of the questionnaire were excluded (*n* = 284). Of the remaining sample with 2,728 participants only people with a GDR socialization [*N* = 1,805; i.e. born before 1980; similar to (48)] were of interest for the following analyses.

### 2.2 Instruments

#### 2.2.1 Psychotherapy experience

Participants were asked whether they had ever experienced psychotherapy in their lives. The frequency of psychotherapy experience was not taken into account. The binary variable was generated using the following question with multiple choice options: ‘Have you ever received psychotherapy as an inpatient in a clinic or as an outpatient, without a stay in a hospital?’ Participants could answer ‘no’, ‘yes, started and already completed’, ‘yes, started and discontinued’ and ‘yes, currently’. For the psychotherapy variable, a distinction was made between ‘no’ and ‘yes’ answers. The question was asked twice: before and after the system change in the GDR to the New Federal States of Germany (the period after reunification). Only yes-answers for the period after the reunification were used as we exploratorily tried to investigate connections between psychotherapy experience and institutional trust after a system change. Information on psychotherapy experience during the GDR can be found elsewhere (49).

#### 2.2.2 Psychological burden

Current anxiety symptoms were recorded using the GAD-7 (Generalized Anxiety Disorder—7) questionnaire. It comprises seven items that can be answered on a 4-point scale from 0 ‘not at all’ to 3 ‘nearly every day’. A sum score (range 0–21) was calculated from this. Higher values indicated more anxiety symptoms. Values ≥ 10 are considered an indication and values ≥ 15 a clear indication of the presence of an anxiety disorder. The questionnaire is a valid and reliable instrument with very good internal consistency [Cronbach’s *α* = 0.89–0.9; (50, 51)]. Somatic symptoms were measured using the SSS-8 (Somatic Symptom Scale—8) questionnaire. It comprises 8 items, which were answered on a 5-point scale from 0 ‘not at all’ to 4 ‘very much’. A total sum score was calculated (range 0–32). A higher score indicates more severe somatic symptoms. Scores from 4 to 7 indicate a low, from 8 to 11 a medium, from 12 to 15 a high and from 16 to 32 a very high somatic symptom burden. The internal consistency (Cronbach’s *α* = 0.81) is good (52). Depressive symptoms were measured by the PHQ-9 (Patient Health Questionnaire—9) questionnaire. It includes nine items that can be answered on a 4-point scale from 0 ‘not at all’ to 3 ‘nearly every day’. A total sum score was formed (range 0–27). The higher the total score, the more depressive symptoms are present. Values of ≥5, ≥10 and ≥15 indicate mild, moderate and severe depression (53). The questionnaire is reliable and valid (53–55) with a very good internal consistency of Cronbach’s *α* = 0.87–0.9 (54, 56).

#### 2.2.3 Experiences of repression during the GDR

The experiences of repression were also collected through self-reporting by the participants. The participants were given a selection of hard and soft forms of repression which were derived from the ‘Social Study of the State of Brandenburg’ (2020) (57)—another representative survey on a regional level of East Germany—and the ‘Thuringian Social Study’ (2008) (58) and slightly adapted. Hard forms of repression were ‘political imprisonment’ and ‘other politically motivated deprivations of liberty’ (score: 0 up to 2); soft forms of repression comprised ‘administrative measures’, ‘school and professional disadvantages’, ‘persecution by the state security or other security organs’, ‘restrictions on personal opinion and freedom of religion and movement’ and ‘afraid of being arrested, watched or limited in the career’ (score: 0 up to 5). Hard and soft forms of repression had to be differentiated according to personally, non-personally (e.g., family members or friends) and not at all experienced.

#### 2.2.4 Institutional trust

Institutional trust was measured with the corresponding questionnaire of the ‘German General Social Survey’ [ALLBUS; Gesis, 2018; (59)]. The questionnaire contains 13 institutions as the parliament or the media for which the participants rated their trust on a 7-point scale (1 no trust at all; 7 very high trust). An average value was calculated across all variables.

#### 2.2.5 Reunification process

The reunification process was covered using three questions of the so called ‘reunification stress index’ (‘Wendebelastungsindex’; WBI) asking for changes in career, finance and privacy on a 4-point scale [improved, worsened, barely changed, incorrect; (60)].

#### 2.2.6 Sociodemographic information, voting intentions and civic engagement

The following sociodemographic items were analyzed: gender (female, male, diverse/other), age, being married and living together with a partner, partnership at all and high school diploma (operationalized as the German school degree ‘Abitur’) and income. Income was measured on a scale comprising 13 subdivisions: 1—up to 500€, 2—500 up to 600€, 3—650 up to 750€, 4—750 up to 900€, 5—900 up to 1,000€, 6—1,000 up to 1,150€, 7—1,150 up to 1,250€, 8—1,250 up to 1,500€, 9—1,500 up to 2,000€, 10—2,000 up to 2,500€, 11—2,500 up to 3,500€, 12—3,500 up to 5,000€, 13—more than 5,000€. Voting intentions were measured as part of the sociodemographic variables. The operationalization of the variable was based on Campbell (37) and indicates whether a person would vote for the German right-wing party ‘Alternative für Deutschland’ (AfD).^1^ This voting behavior is generally thought to be an indication of dissatisfaction with the existing political system (61). Civic engagement was measured using the items of the German Socioeconomic Panel (SOEP) 2018 and 2009 (62) surveys with lower levels indicating more engagement. A total sum value was calculated from the reported commitment in leisure time to get involved in (a) political parties or citizens’ initiatives as well as in (b) clubs or associations (62). The frequency of both items (a and b) could be specified as 1 (every week), 2 (every month), 3 (less often) or 4 (never).

### 2.3 Statistical analysis

For the investigation of psychotherapy use, multiple logistic regression models were constructed, whereas we calculated multiple linear regression models for the investigation of institutional trust. The assumptions for logistic regression were fulfilled. The observations were independent, the scale level of therapy experience is binary. Only the SSS-8, GAD-7 and PHQ-9 strongly correlated (*r* < 0.700), but were added simultaneously. The assumptions of linear regression models were also fulfilled. There were no autocorrelation and multicollinearity except for the WBI questions concerning career and finance (*r* = 0.516). Accordingly, the career variable was excluded. Homoscedasticity and normal distribution of the residuals were assured. The Breusch Pagan test (63, 64) and the White test (65) on heteroskedasticity were not significant.

First, a basic regression model for psychotherapy use (including the predictors female gender, high school diploma, not living together with a partner, income and the sum scores of GAD-7, SSS-8 and PHQ-9) was investigated. In a next step, experiences of repression were added before the basic model to check further explanation of variance.

Then a multiple linear model was calculated for institutional trust following the procedure of Campbell (37) for the three groups distinguished by having no/non-personal/personal experience of repression. Separate, but identical basic models were built for the three groups. Those included the following predictors: female gender, age, high school diploma, married and living together, income, civic engagement, voting AfD and the WBI questions for financial and private changes concerning the reunification. Afterwards the factor therapy experience was added to explore a link between psychotherapy experience and institutional trust. All calculations were performed using the statistical software SPSS (version 21). Both regression analyses were carried out with GENLIN. *φ* (2*2 contingency tables) and Cramér’s V (>2*2 contingency tables) were interpreted according to Cohen’s criteria (66) with 0.1 indicating small, 0.3 medium and 0.5 large effect sizes (absolute value in case of *φ*). ANOVA was calculated to measure group differences for interval scaled measures. Kruskal-Wallis-Tests were calculated when the assumption of normal distribution or equal variances were not fulfilled. In all analyses, people who selected the answer option ‘I do not know’ of an item or denied answering an item at all, were excluded of the specific analysis.

### 2.4 Ethics approval

This study was conducted by the principles of good scientific practice and was approved by the Ethics Committee of the University Hospital of Leipzig on the 28th of March 2022 (file number 091/22-ek). Participation was voluntary and anonymous. The participants provided informed consent and had a right to withdraw from the study.

## 3 Results

### 3.1 Characteristics of the study population with experiences of repression

On average, slightly more women than men took part in the study; the age was about 61 years. Most of them lived with a partner, 12% had undergone psychotherapy after the reunification process. On average, an income of around €1,150–1,500 was reported and the sample achieved the cut-off value for the presence of somatic symptom distress. 44% were affected by repression (personally or non-personally). More than half of the participants were married and lived together with their partner (Table 1).

**Table 1:** Sample description.

Variables	GDR socialized sample (*n*_max_ = 1,805)
Female gender	*n* = 1,010 (56.0%)
Age	*m* = 61.25 (*sd* = 10.83)
High school diploma	*n* = 468 (26%)
Being married and living together with partner	*n* = 971 (54%)
Therapy experience	*n* = 222 (12%)
Income	*m* = 7.77 (*sd* = 2.66)
GAD-7	*m* = 2.58 (*sd* = 2.72)
PHQ-9	*m* = 3.81 (*sd* = 3.85)
SSS-8	*m* = 5.64 (*sd* = 4.76)
Experienced repression	
No	*n* = 1,015 (56%)
Non-Personally	*n* = 523 (29%)
Personally	*n* = 267 (15%)

Percentages in brackets refer to the relative frequency in the subgroup.

Men reported more personal experience of political imprisonment, persecution, restrictions and fear of further persecution., but the effect sizes were small (Table 2).

**Table 2:** Personal experienced forms of governmental misuse of power and gender.

Variable	*N*	Men	Female	*p-*value	*φ*
Political imprisonment	*N* = 1,805	*n* = 795	*n* = 1,010	*p* < 0.001***	−0.1
Yes		16 (2%)	1 (<1%)		
No		779 (98%)	1,009 (<100%)		
Other politically motivated deprivations of liberty	*N* = 1,805	*n* = 795	*n* = 1,010	*p* = 0.323	–
Yes		9 (1%)	7 (1%)		
No		786 (99%)	1,003 (99%)		
Administrative measures	*N* = 1,805	*n* = 795	*n* = 1,010	*p* = 0.323	–
Yes		9 (1%)	7 (1%)		
No		786 (99%)	1,003 (99%)		
School and professional disadvantages	*N* = 1,805	*n* = 795	*n* = 1,010	*p* = 0.051	–
Yes		66 (8%)	60 (6%)		
No		729 (92%)	950 (94%)		
Persecution by the state security or other security organs	*N* = 1,805	*n* = 795	*n* = 1,010	*p* = 0.002**	−0.07
Yes		45 (6%)	28 (3%)		
No		750 (94%)	982 (97%)		
Restrictions on personal opinion and freedom of religion and movement	*N* = 1,805	*n* = 795	*n* = 1,010	*p* = 0.005**	−0.07
Yes		77 (10%%)	62 (6%)		
No		718 (90%)	948 (94%)		
Afraid of being arrested, watched or limited in the career	*N* = 1,805	*n* = 795	*n* = 1,010	*p* = 0.002**	−0.07
Yes		79 (10%)	60 (6%)		
No		716 (90%)	950 (94%)		

Chi square tests (Fisher Exact in cases of small sample size < 5) were calculated. **p < 0.01, ***p < 0.001.

**Figure 1:**
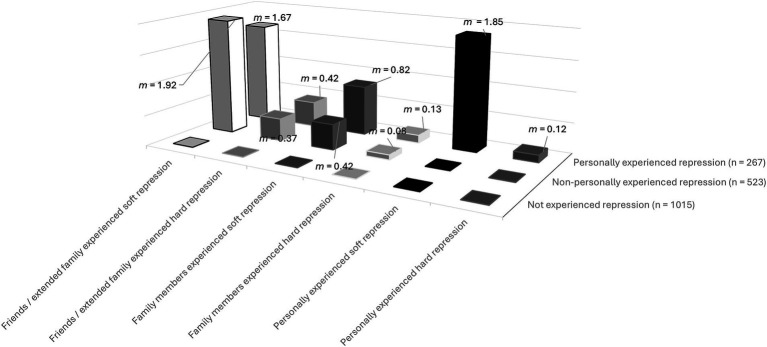
Mean number of experiences of repression according to group assignment (not/non-personally/personally experienced repression). Y-axis: mean number of experiences of repression; x-axis: group assignment; z-axis: hard and soft forms of repression by proximity (personally, family, friends and extended family).

Figure 1 shows the mean number of experienced repressions in the groups no, non-personally and personally experienced repression of the by repression affected participants and the degree of proximity (personally/family/friends and extended family)—more proximity often goes hand in hand with more experiences of repression at all.

### 3.2 Psychotherapy experience

In the basic model, all variables—except partnership, income and somatic symptoms—were significant predictors of psychotherapy experience (Table 3). The singularly added variable experienced repression was significant in the extended model, with personal experiences of repression having a more significant impact than non-personal experiences. The same variables were statistically significant as in the basic model, except high school diploma which lost significance (Table 3).

**Table 3:** Logistic regression on therapy experience—basic and extended models.

Variable of the model	*N* (Omnibus test)	Multiple linear analysis	
		OR (95% CI)	*p*-value
Constate term	1,739 (*X^2^* = 120.19; df = 8; *p* < 0.001***)	0.135 (0.048; 0.383)	<0.001***
Female gender		2.556 (1.818; 3,595)	<0.001***
Age		0.975 (0.961; 0.989)	0.001**
High school diploma		1.515 (1.073; 2.139)	0.018*
No partnership		1.177 (0.857;1.616)	0.314
Income		1.002 (0.946; 1.061)	0.956
GAD-7		1.122 (1.052; 1.196)	<0.001***
SSS-8		1.018 (0.978; 1.059)	0.377
PHQ-9		1.067 (1.016; 1.120)	0.010*
Constate term	1,739 (*X^2^* = 144.58; df = 10; *p* < 0.001***)	0,113 (0.039; 0.328)	0.078
Experienced repression			<0.001***
Personally		2.766 (1.852; 4.131)	<0.001***
Non-personally		1.570 (1.103; 2.235)	0.012*
Not		Ref.	-
Female gender		2.768 (1.957; 3.917)	<0.001***
Age		0.974 (09.59; 0.988)	<0.001***
High school diploma		1.355 (0.954; 1.926)	0.090
No partnership		1.157 (0.840;1.594)	0.372
Income		0.996 (0.939; 1.056)	0.890
GAD-7		1.104 (1.034; 1.180)	0.003**
SSS-8		1.016 (0.976; 1.058)	0.439
PHQ-9		1.068 (1.016; 1.123)	0.010*

The additional factor experienced repression was added to the basic model. *p < 0.05, **p < 0.01, ***p < 0.001.

### 3.3 Trust values

**Figure 2:**
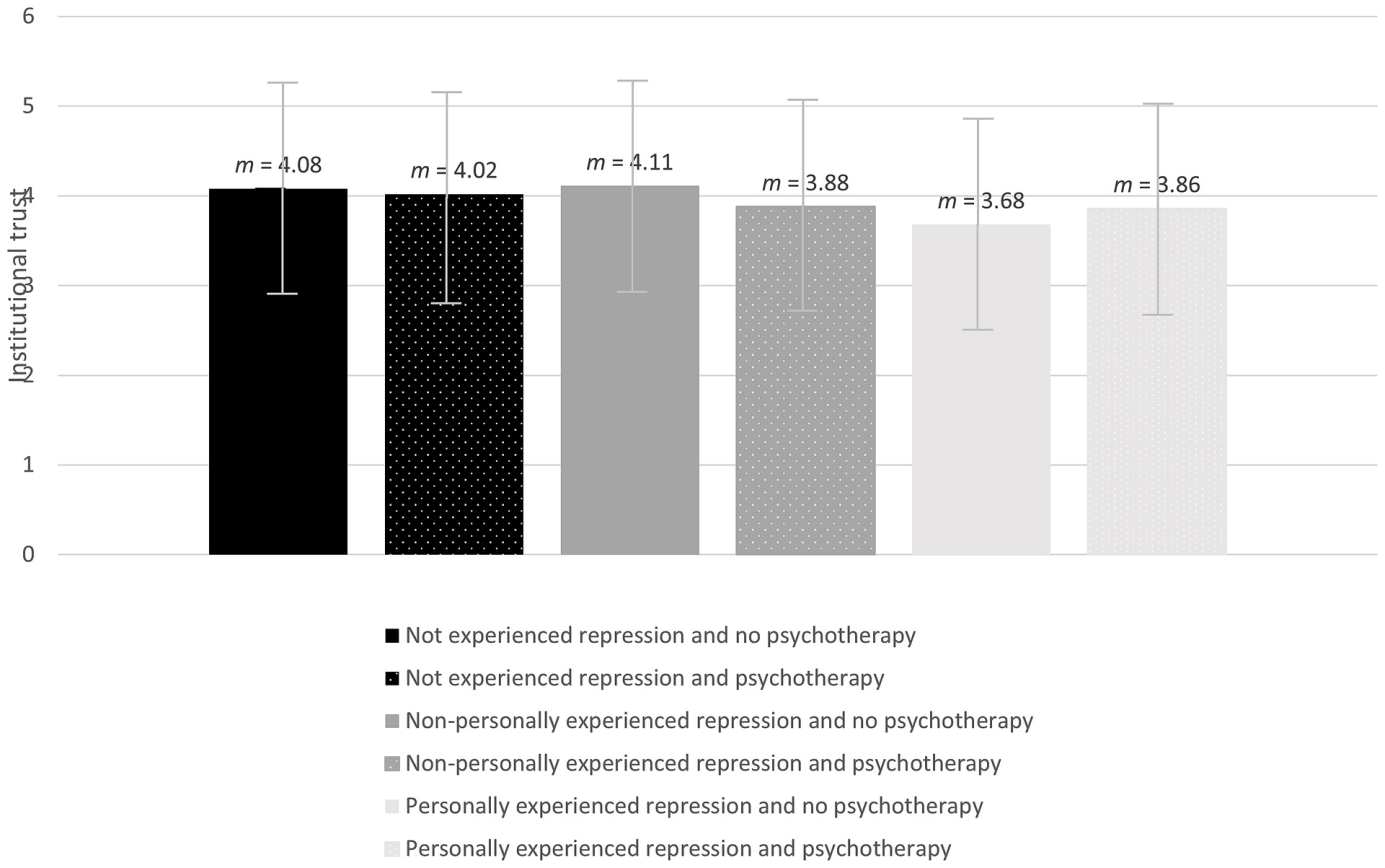
Trust values according to experiences of repression and psychotherapy.

Figure 2 shows different trust values of the three repression groups and their psychotherapy experience. Participants who mentioned personally experienced repression reached lowest trust scores.

The groups differed in almost all variables of the basic model (Table 4). Therefore, and to determine specific group effects, we calculated the models separately for the three groups. Similar to the analysis strategy for institutional trust by Campbell (37) we conducted linear regression models.

**Table 4:** Differences between the three groups according to the variables in the linear regression model.

Variable of the model	Not experienced repression (*n*_max_ = 1,015)	Non-personally experienced repression (*n*_max_ = 523)	Personally experienced repression (*n*_max_ = 267)	*p*-value	Cramér’s *V*/*X*^2^
Female gender	*n* = 594 (59%)	*n* = 293 (56%)	*n* = 123 (46%)	0.001**	0.09
^1^Age	*m* = 61.02 (*sd* = 11.15)	*m* = 61.21 (*sd* = 10.67)	*m* = 62.23 (*sd* = 9.84)	0.194	–
High school diploma	*n* = 231 (23%)	*n* = 140 (27%)	*n* = 97 (37%)	<0.001***	0.11
Married and living together	*n* = 554 (55%)	*n* = 288 (55%)	*n* = 129 (48%)	0.148	–
^1^Income	*m* = 7.62 (*sd* = 2.71)	*m* = 7.99 (*sd* = 2.46)	*m* = 7.93 (*sd* = 2.83)	0.024*	8.020
Civil engagement	*m* = 7.24 (*sd* = 1.20)	*m* = 6.80 (*sd* = 1.41)	*m* = 6.64 (*sd* = 1.54)	<0.001***	58.946
^2^Voting AfD	*n* = 76 (8%)^3^	*n* = 49 (9%)^4^	*n* = 34 (13%)^5^	0.024*	0.07
WBI—finance				0.133	–
Improved	*n* = 531 (53%)	*n* = 300 (58%)	*n* = 154 (59%)		
Worsened	*n* = 180 (18%)	*n* = 81 (16%)	*n* = 48 (18%)		
Barely changed	*n* = 241 (24%)	*n* = 114 (22%)	*n* = 56 (21%)		
Incorrect	*n* = 54 (5%)	*n* = 24 (5%)	*n* = 5 (2%)		
WBI—private				0.086	–
Improved	*n* = 159 (16%)	*n* = 92 (18%)	*n* = 43 (16%)		
Worsened	*n* = 147 (15%)	*n* = 90 (17%)	*n* = 51 (19%)		
Barely changed	*n* = 643 (64%)	*n* = 316 (61%)	*n* = 163 (62%)		
Incorrect	*n* = 57 (6%)	*n* = 20 (4%)	*n* = 6 (2%)		
Therapy experience	*n* = 91 (9%)	*n* = 74 (14%)	*n* = 57 (21%)	<0.001***	0.13

Chi square tests and ^1^Kruskal-Wallis-Tests were calculated. *p < 0.05, **p < 0.01, ***p < 0.001. Percentage in brackets refers to the relative proportion in the subgroup. Before we asked the participants which party they would vote for, we asked them whether they would vote at all. ^2^The relative proportion of AfD-voters refers to those who stated that they would go to the polls next Sunday. Voting intensions indicated ^3^71%, ^4^82% and ^5^81%.

In all three groups in the basic model, voting AfD and WBI finance were relevant. Voting AfD was associated with lower levels of institutional trust, participants who reported a worsened financial situation since the reunification had lower levels of institutional trust in comparison with participants who reported an improved financial situation. Only in the group which did not report repression, the variables being married and living together and WBI private were statistically significant. Being married and living together was related to higher levels of institutional trust in this group. Participants who reported a worsened private situation or answered that none of the response options could describe their private situation since the reunification had lower levels of institutional trust in comparison with participants who reported an improved private situation. Finally, the binary psychotherapy variable was added into the model which was only statistically significant in the group with participants who personally experienced repression. In this case, psychotherapy was related to higher values of institutional trust. Voting AfD and WBI finance remained relevant for institutional trust (Table 5).

**Table 5:** Linear regressions on institutional trust for the three groups-basic and extended models.

Variable of the model	Multiple linear model for participants with no experienced repression (*n* = 963; *X*^2^ = 118.73; df = 13; *p* < 0.001***)		Multiple linear model for participants with non-personally experienced repression (*n* = 498; *X*^2^ = 50.01; df = 13; *p* < 0.001***)		Multiple linear model for participants with personally experienced repression (*n* = 247; *X*^2^ = 66.98; df = 13; *p* < 0.001***)	
	Adjusted OR (95% CI)	*p*-value	Adjusted OR (95% CI)	*p*-value	Adjusted OR (95% CI)	*p*-value
Constate term	52.615 (25.873; 106.995)	<0.001***	60.530 (25.579; 143.238)	<0.001***	33.374 (9.408; 118.383)	<0.001***
Female gender	1.088 (0.939; 1.262)	0.261	0.968 (0.792; 1.183)	0.748	0.926 (0.700; 1.224)	0.590
Age	0.998 (0.991; 1.004)	0.522	1.009 (0.999; 1.018)	0.089	1.012 (0.998; 1.027)	0.085
High school diploma	0.939 (0.787; 1.120)	0.482	1.055 (0.840; 1.325)	0.645	1.204 (0.903; 1.607)	0.206
Married and living together	1.334 (1.153; 1.544)	<0.001***	1.174 (0.966; 1.426)	0.107	1.034 (0.781; 1.369)	0.813
Income	1.015 (0.985; 1.045)	0.330	1.012 (0.970; 1.057)	0.575	0.992 (0.939; 1.048)	0.782
Civic engagement	0.979 (0.922; 1.040)	0.488	0.939 (0.877; 1.006)	0.074	0.958 (0.876; 1.046)	0.337
Voting AfD	0.390 (0.298; 0.511)	<0.001***	0.532 (0.381; 0.742)	<0.001***	0.302 (0.201; 0.454)	<0.001***
WBI—finance		0.012*		0.003**		<0.001***
Improved	Ref. 1		Ref. 1		Ref. 1	
Worsened	0.765 (0.617; 0.947)	0.014*	0.576 (0.430; 0.771)	<0.001***	0.382 (0.254; 0.573)	<0.001***
Barely changed	0.835 (0.696; 1.002)	0.52	0.919 (0.714; 1.183)	0.513	0.887 (0.627; 1.254)	0.497
Incorrect	0.652 (0.459; 0.926)	0.017*	0.930 (0.547; 1.583)	0.790	0.807 (0.304; 2.141)	0.666
WBI—private		<0.001***		0.231		0.548
Improved	Ref. 1		Ref. 1		Ref. 1	
Worsened	0.883 (0.674; 1.156)	0.365	0.713 (0.505; 1.006)	0.054	0.974 (0.606; 1.566)	0.913
Barely changed	1.417 (1.152; 1.741)	0.001***	0.881 (0.671; 1.157)	0.363	1.210 (0.819; 1.788)	0.338
Incorrect	1.497 (1.031; 2.173)	0.034	0.727 (0.391; 1.354)	0.315	0.949 (0.378; 2.383)	0.912
	(*n* = 963; *X^2^* = 118.79; df = 14; *p* < 0.001***)		(*n* = 498; *X^2^* = 50.57; df = 14; *p* < 0.001***)		(*n* = 247; *X^2^* = 71.20; df = 14; *p* < 0.001***)	
Constate term	52.207 (25.606; 106.441)	<0.001***	61.986 (26.148; 146.929)	<0.001***	29.139 (8.249; 102.932)	<0.001***
Psychotherapy experience	1.032 (0.805; 1.324)	0.803	0.898 (0.680; 1.187)	0.451	1.411 (1.017; 1.957)	0.039*
Female gender	1.085 (0.935; 1.260)	0.284	0.975 (0.797; 1.193)	0.806	0.882 (0.666; 1.168)	0.381
Age	0.998 (0.991; 1.004)	0.530	1.008 (0.998; 1.018)	0.097	1.013 (0.999; 1.028)	0.062
High school diploma	0.939 (0.787; 1.120)	0.486	1.062 (0.845; 1.335)	0.603	1.196 (0.898; 1.592)	0.220
Married and living together	1.335 (1.153; 1.545)	<0.001***	1.166 (0.958; 1.418)	0.125	1.077 (0.814; 1.426)	0.603
Income	1.015 (0.986; 1.045)	0.326	1.012 (0.970; 1.056)	0.585	0.992 (0.940; 1.048)	0.776
Civic engagement	0.979 (0.922; 1.040)	0.498	0.939 (0.877; 1.006)	0.073	0.962 (0.881; 1.051)	0.391
Voting AfD	0.391 (0.298; 0.511)	<0.001***	0.530 (0.380; 0.740)	<0.001***	0.300 (0.200; 0.450)	<0.001***
WBI—finance		0.012*		0.003**		<0.001***
Improved	Ref. 1		Ref. 1		Ref. 1	
Worsened	0.765 (0.617; 0.948)	0.014*	0.577 (0.431; 0.773)	<0.001***	0.370 (0.247; 0.554)	<0.001***
Barely changed	0.835 (0.696; 1.002)	0.53	0.917 (0.713; 1.181)	0.503	0.858 (0.607; 1.212)	0.384
Incorrect	0.651 (0.458; 0.925)	0.017*	0.935 (0.550; 1.592)	0.806	0.720 (0.272; 1.907)	0.509
WBI—private		<0.001***		0.246		0.635
Improved	Ref. 1		Ref. 1		Ref. 1	
Worsened	0.881 (0.672; 1.154)	0.358	0.717 (0.508; 1.012)	0.059	0.977 (0.610; 1.565)	0.924
Barely changed	1.417 (1.153; 1.742)	0.001***	0.886 (0.675; 1.165)	0.387	1.186 (0.805; 1.747)	0.389
Incorrect	1.497 (1.031; 2.173)	0.034	0.739 (0.397; 1.376)	0.340	0.950 (0.381; 2.365)	0.911

The additional factor psychotherapy was added to the basic model. *p < 0.05, **p < 0.01, ***p < 0.001.

## 4 Discussion

Our study revealed an estimation of 15% personally by repression affected persons who lived in the former GDR with a dictator regime. Further 29% reported an indirect, non-personal affection. The prevalence of psychotherapy usage after the reunification ranged from 9% (not affected by repression) to 21% (personally affected by repression) with a mean of 12%—indicating less psychotherapy usage than in complete Germany (6). Repression was highly correlated with psychotherapy use in our analysis. Consequently, for people affected by repression, previously well-known factors as psychological symptoms or gender do not seem sufficient to explain psychotherapy usage. Persons who reported being affected by repression showed lower levels of institutional trust. Our study exploratorily investigated connections between psychotherapy usage after a system change and institutional trust. Our results show that psychotherapy is associated with higher levels of institutional trust among people who have personally experienced repression in comparison with people who have personally experienced repression but have not had psychotherapy. Psychotherapy might thus have a reconciling function for experiences of injustice and mistrust, but due to correlational analysis strategy causal attributions are not possible. It is also conceivable that people with greater trust in institutions are more willing to undergo psychotherapy. Furthermore, we identified significant group differences between the three groups (personally, non-personally, not at all by repression affected) that may have influenced the results on institutional trust. For example, the proportion of women in the group of those personally affected was significantly lower. The level of education was also higher here.

It is important for psychotherapists to have knowledge of the fact that people with experiences of repression in East Germany or maybe also in other post-authoritarian systems are particularly likely to seek psychotherapy after a system change. Consequently, training programs for psychotherapists that address experiences of repression are important. Furthermore, patients with experiences of repression are often characterized by a lack of trust in institutions. Psychotherapists should be aware that mistrust can be related to experiences of repression. It is important to recognize that mistrust can also relate to other institutions, not necessarily the healthcare system: This patient group requires support in learning when and in what they can trust. For these clients it can be important that psychotherapists create a trusting framework in psychotherapy. The present study indicates a correlation between higher levels of institutional trust and the experience of psychotherapy. Further research is required on how to build trust in people who have experienced repression. In other fields it is known that e.g. psychoeducation can help rebuilding trust in psychotherapy (67) or psychotherapeutic openness is recommended for trust (68). Whether these or other factors in psychotherapy are relevant in building institutional trust in persons affected by repression should be examined in the future. The findings also have policy implications for mental health in post-authoritarian systems. The findings provide initial evidence that the structural determinants of mental health need to be addressed in order to restore trust in healthcare systems in populations that have experienced repression.

Regarding repression forms it has to be considered that men seem to be more often personally affected. It also has to be taken into account that the forms of repression have changed over time, and we mainly received information related to the last decades of the GDR. Furthermore, we have to expect a survival selection in our sample (69–71). Interestingly, the category being afraid of being observed, was only rarely selected. Observations may have been an everyday phenomenon. Awareness of the full extent of observation probably became known after the reunification or being afraid was not a feeling evoked by the possibility of observation.

Persons who experienced repression more often reported higher levels of education which was surprising as opponents often faced restrictions to education. Their income was also higher than the income of the participants without experiences of repression. The question arises whether education or socio-economic factors could possibly influence the reporting of repression. It is more likely that they became oppositional after their education. Studies on oppositional behavior and education could continue to be informative here. Previous research shows that higher education is associated with lower institutional trust in corrupt countries and with higher institutional trust in non-corrupt countries (72). In addition, people trust authoritarian states more if they are doing well economically or if they themselves share authoritarian attitudes (73, 74). Furthermore, the undermining of interpersonal trust seems to be relevant for the prevention of revolts in authoritarian states (75). The links between psychotherapy, institutional trust and interpersonal trust need to be further clarified. Also, the question arises as to whether these results could be relevant in regions with persistent repression. Here it could be important whether the psychotherapeutic facilities are state or independent institutions. Besides, no distinction was made between the duration and frequency of experiences of repression. More differentiated analyses in the future may provide further insights here. Further research on these issues is needed as a general, international lack concerning experiences of trust, repression and the rebuilding of health systems can be seen.

### 4.1 Limitations

This study was able to identify a link between the three factors repression, psychotherapy and institutional trust with a potential trust reconciling function of psychotherapy. Due to the nature of the study, we were not able to make causal attributions. We also specified and asked about the categories of experiences of repression. There was no option for specifying additional or different forms of repression. In addition, the specific circumstances of the psychotherapy experience, which may be important, were unknown. We identified significant group differences between the three groups (personally, non-personally, not at all by repression affected) that may have influenced the results on institutional trust. Furthermore, we did not take internal migration into account. This might be relevant for our results, since migrating to the West—especially if it occurred longer ago in a period of greater differences between East and West—may have significant effects on socioeconomic parameters as job opportunities and consequently income as well as other environmental factors as the access to healthcare institutions including psychotherapy that interfere with our analyses (76). These factors may independently influence both psychotherapy usage and institutional trust. But this group of internal migrants is very difficult to identify due to its small number in the population (77). Furthermore, the data is based exclusively on self-reporting. Nevertheless, self-reporting is a viable method for collecting sensitive personal experiences on a large scale, where privacy and ethical considerations are very important. The results could also be relevant for other regions where people are experiencing or have experienced repression. However, access to mental health services and cultural factors affecting attitudes to psychotherapy (49) need to be taken into account.

### 4.2 Conclusion

This study investigated the connections between experiencing past repression, psychotherapy experience after a system change and current institutional trust in a sample of 1,805 individuals living in East Germany who were also socialized in the GDR. A connection was found between the three factors. We could show that experiencing repression is a crucial factor for predicting psychotherapy experience, and psychotherapy experience is associated with an increased institutional trust in persons who are personally affected by repression. This indicates that psychotherapy might play a role in both containing the personal suffering after experiencing repression and reestablishing institutional trust after a system change. Psychotherapists should be aware that people who have experienced repression are a key client group in East Germany. Knowledge of repression and an awareness of the inherent mistrust of those affected by repression should be taken into account in psychotherapy. Practitioners who wish to specialize in these clients should, for example, be familiar with the procedures and laws of rehabilitation (78). In addition, it could be important to support clients in such application procedures that go through institutions because these clients may experience mistrust toward these institutions, which should be addressed and dealt with in therapy. The findings also provide initial evidence that the structural determinants of mental health need to be addressed to restore trust in healthcare systems in populations that have experienced repression.

## Data Availability

The raw data supporting the conclusions of this article will be made available by the authors by reasonable request.

## Footnotes

^1^The party was founded in 2013 and is the first established party on the far right of the party system in the Federal Republic of Germany.
